# Single-exposure messages for child sexual abuse prevention: a randomized controlled online trial testing educational and humanizing approaches

**DOI:** 10.1186/s40359-026-04829-3

**Published:** 2026-05-23

**Authors:** Viola Westfal, Maximilian von Heyden

**Affiliations:** Charité – Universitätsmedizin Berlin, corporate member of Freie Universität Berlin and Humboldt-Universität Zu Berlin, Institute of Sexology and Sexual Medicine, Charitéplatz 1, Berlin, 10117 Germany

**Keywords:** Child sexual abuse prevention, Child sexual abuse material, Public health communication, Stigma reduction, Psychoeducation, Humanizing interventions, Health messaging

## Abstract

**Background:**

Child sexual abuse and exploitation, including online material depicting such acts, constitute a major public health concern. Prevention efforts likely benefit from public understanding and destigmatization, yet communication strategies on this topic face persistent misconceptions and strong emotional reactions. This randomized controlled online study examined whether brief educational and humanizing communication interventions influenced knowledge, attitudes, and behavioral intentions related to prevention, while avoiding adverse emotional or ethical effects.

**Methods:**

A total of 2,019 adults from Spain and Portugal were recruited via an online panel and randomly assigned to one of four conditions: an educational message, a humanizing message with empathy focus, a humanizing message with prevention focus, or a neutral control message. Each intervention consisted of a short video and accompanying text presented as part of a health information campaign. Measures included affective state, stigma-related attitudes, literacy about therapeutic options, openness toward the topic, and behavioral intention to seek information.

**Results:**

Exposure to any intervention message led to short-term changes in affective responses, with lower positive valence and slightly higher arousal compared to the control group (medium effect for valence, small for arousal). No significant differences emerged for stigma dimensions, knowledge, openness, or behavioral intentions. All three intervention formats performed similarly. The study did not identify measurable short-term adverse or rebound effects on stigma. Dropout rates did not differ across conditions, but languages.

**Conclusions:**

Brief communication interventions elicited short-term affective responses without evidence of increased stigma or avoidance on the measured outcomes. While the present single exposures appear insufficient to change attitudes or behavior, such formats may serve as a foundation for repeated, multi-channel prevention messaging integrated into broader educational efforts.

**Trial registration:**

German Clinical Trials Register (DRKS), DRKS00038927, retrospectively registered on 09/01/2026.

## Background

Child sexual exploitation and abuse, especially online, is increasingly recognized as a global public health concern. A recent meta-analysis by Fry and colleagues [15] found that approximately 12.5% of children experienced online sexual exploitation within the last year; child lifetime exposure was found to have a prevalence of 11.5%. However, child sexual abuse is considered as preventable, particularly through early intervention, education, and effective policy implementation [13].

In order to understand how prevention efforts can be implemented, it is necessary to first clarify the key concepts central to this discussion: According to the World Health Organization [55], child sexual abuse (CSA) is “the involvement of a child in sexual activity that he or she does not fully comprehend, is unable to give informed consent to, or for which the child is not developmentally prepared and cannot give consent, or that violates the laws or social taboos of society. Child sexual abuse is evidenced by this activity between a child and an adult or another child who by age or development is in a relationship of responsibility, trust or power, the activity being intended to gratify or satisfy the needs of the other person.” The term *“child sexual abuse material”* (CSAM) is increasingly adopted in place of *“child pornography”*, as it more accurately reflects the abusive and exploitative nature of such content. The rationale behind this shift in terminology is that material depicting or representing sexual acts involving children constitutes a form of child sexual abuse, regardless of the medium or intent, and should not be trivialized by the term “pornography” [51]. CSAM is typically used to refer to a subset of *child sexual exploitation material* (CSEM) that explicitly involves actual abuse or focuses on the anal or genital regions of the child [51].

*Pedophilia*, in basic terms, refers to a sexual interest in prepubescent children. Clinically, it is only considered a disorder (*pedophilic disorder*) when this interest is intense, persistent, and focused, typically manifested through recurring sexual thoughts, fantasies, urges, or behaviors involving children who have not yet reached puberty. According to the *Diagnostic and Statistical Manual of Mental Disorders, Fifth Edition (DSM-5)*, a diagnosis of pedophilic disorder requires that the individual is at least 16 years old, the child of interest is at least five years younger, and that the individual has either acted on these urges or experiences significant distress because of them [2]. While the existence of a pedophilic sexual preference or pedophilic disorder is by no means equivalent to committing CSA/M, pedophilia or pedophilic disorder is nevertheless considered a risk factor for sexual offenses involving children [48].

Although these groups overlap conceptually and may share barriers related to stigma, secrecy, and help-seeking, they are neither clinically nor behaviorally equivalent. The present study therefore uses the umbrella term “*individuals at risk*” deliberately in a broad public health communication sense. One aim of prevention-oriented public health messaging is to raise awareness of the existence and accessibility of preventive support services for individuals who may experience risk-related concerns, regardless of their specific diagnostic status or offense history.

At the level of the intervention materials, however, the communication focus is more narrowly operationalized around the use of CSAM as a concrete and clearly identifiable risk behavior. This behavioral focus was chosen because CSAM use may occur in heterogeneous populations and for different underlying reasons, without requiring prior diagnostic classification. The intervention messages therefore address prevention and help-seeking in relation to CSAM-related risk behavior while acknowledging the broader heterogeneity of populations potentially targeted by prevention efforts.

Although CSAM is a growing concern within global child protection efforts, the development and implementation of effective prevention strategies face challenges. One of these are public perception and responses related to CSA and CSAM: Research suggests that CSA offenses elicit stronger emotional reactions than other crimes, even murder [44]. This can lead to an inaccurate assessment of risk and policy decision making undermining evidence-based practices [13, 16, 25]. The public conflation of pedophilia with the consumption of CSAM reinforces stigmatization [30, 46]. As a result, individuals who consume or are at risk of consuming CSAM may experience shame and fear of judgment, both of which are known to hinder help-seeking behavior [30].

### Stigma in the context of CSA/M prevention

Stigma theory, as formulated by Link and Phelan [36] provides a crucial framework for understanding the barriers to prevention efforts in CSA/M contexts. Stigma operates through a multi-level process: first through public stigma, which subsequently becomes internalized as self-stigma among affected individuals [9, 36].

In the context of individuals at risk of CSA/M, this stigmatization process creates a significant barrier to prevention and treatment. Research specific to this population demonstrates that individuals attracted to children who internalize stigmatizing attitudes experience heightened shame, depression, and anxiety [18]. Critically, this internalized stigma directly impairs help-seeking behavior. As Grady and colleagues [18] found in their study internalized stigma, fear of being reported, and negative reactions from therapists concerns represent major barriers preventing individuals from seeking mental health support.

This creates a paradoxical situation that undermines evidence-based prevention strategies. While society aims to prevent child sexual abuse through deterrence and punishment, stigmatization reduces the likelihood that individuals at risk will voluntarily seek help before engaging in harmful behavior [44]. As Lawrence and Willis [32] note, “anti-stigma interventions are therefore an important component of public health approaches to the prevention of child sexual abuse, as well as an important strategy for reducing adverse outcomes among nonoffending individuals living with a sexual interest in children”. For the general public, anti-stigma interventions serve to correct misconceptions about pedophilia and CSAM users, distinguish between sexual attraction and sexual behavior, and foster understanding of prevention and treatment possibilities. For individuals at risk, anti-stigma interventions aim to reduce anticipated stigma that prevents help-seeking, increase awareness of available treatment and support services, and create a social environment supports treatment engagement.

Reducing public stigma may constitute one relevant component of broader prevention-oriented public health strategies. The mechanism is indirect but theoretically well-grounded: when the general public holds more accurate and less dehumanizing views of individuals with pedophilic interests, the anticipated social cost of disclosure decreases, which may contribute to lowering psychological barriers to help-seeking among those at risk [9, 18]. In other words, public attitudes shape the social environment within which individuals at risk decide whether to seek professional support, making the general public a legitimate and necessary target for prevention-oriented communication. Public health messaging campaigns aimed at the general population are therefore a scalable mechanism for shifting this social environment: they can correct misinformation, reduce dehumanizing perceptions, and foster public support for rehabilitative rather than purely punitive responses. Despite the clear theoretical rationale for such campaigns, the empirical evidence base remains limited. Most existing studies rely on student samples, English-speaking populations, or single-country designs, and few have tested communication strategies under randomized controlled conditions. The present study directly addresses this gap.

### Communication mechanisms of stigma reduction

Research on stigma reduction and attitude change has identified two broad mechanisms through which communication interventions can alter public perceptions of stigmatized groups: an *informational-cognitive route*, operating through knowledge correction and stereotype reduction, and an *affective-narrative route*, operating through empathy activation, perspective-taking, and humanization [9, 19].

These two mechanisms align with dual-process models of persuasion [19], which distinguish between message processing driven by the elaboration of factual content and processing driven by narrative immersion and emotional engagement. The present study tests three communication interventions designed to target these mechanisms: an educational message targeting the informational-cognitive route (intervention 1, IN1), a humanizing empathy-focused narrative targeting the affective-narrative route through perspective-taking and emotional engagement (intervention 2, IN2), and a humanizing prevention-focused narrative operating as a hybrid of both routes, combining narrative humanization with perceived self-regulation and cognitive elaboration of perceived behavioral controllability (intervention 3, IN3).

(Psycho)educational interventions operate primarily through the informational-cognitive route: They aim to close the gap between expert knowledge and public understanding by correcting misinformation and replacing stigma-sustaining stereotypes with accurate scientific information. This approach is grounded in the assumption that stigma persists due to misinformation and stereotype activation [9]. Research has demonstrated that psychoeducational interventions can shift attitudes towards people who committed sexual offenses [56, 57], people with a sexual interest in children [20, 21, 29, 31, 39], and may potentially influence perceptions of CSAM-related behavior as well [49]. The educational intervention in our study provides evidence-based information about pedophilia, the distinction from child sexual abuse, information about treatment possibilities, and the reality that not all individuals with such interests commit offenses. This was designed to address the public's knowledge gaps while remaining grounded in scientific evidence.

Humanizing interventions operate through the affective-narrative route. Rather than providing factual arguments, they present individualized, story-based depictions of members of a stigmatized group that highlight their uniquely human qualities like emotional complexity, inner struggles, and capacity for self-reflection and moral agency [23]. Dehumanization – the denial of these uniquely human attributes to members of a stigmatized group – has been proposed as one mechanism contributing to stigma formation [23, 36]. In the context of CSA/M, public discourse frequently employs dehumanizing language such as “monsters” or “predators” [22, 32], which reinforces the perception that individuals with a sexual interest in children are categorically different from and more threatening than typical people, sustaining exclusionary attitudes and obstructing help-seeking. By providing tangible and accurate depictions of non-offending individuals with a sexual interest in children, humanizing narratives can lead to an improvement in public attitudes [21, 22, 31].

Experimental evidence supports the effectiveness of humanizing information in changing intergroup attitudes and behavior. Borinca, McAuliffe, and Nightingale [5] demonstrated across two studies that humanizing information – compared to equally positive but non-humanizing information – improved behavioral intentions toward stigmatized outgroup members by reducing intergroup anxiety and increasing empathy. Importantly, these effects were moderated by prior negative contact: humanizing information was most beneficial for individuals who reported higher levels of negative prior contact with the outgroup, suggesting that such messages may be particularly impactful for the most resistant audiences, provided they successfully activate the relevant affective pathways.

The Stereotype Content Model (SCM; [12]) provides a structural framework for understanding the stigma profile that humanizing messages must address. The SCM proposes that social groups are primarily evaluated along two dimensions: *warmth* (perceived benevolence and prosocial intent) and *competence* (perceived capability). Groups perceived as low in warmth and high in perceived threat elicit contempt and fear; a configuration that accurately describes how individuals with sexual interest in children are typically perceived. Humanizing narratives that convey emotional complexity and inner moral struggle may shift warmth perceptions; however, if perceived dangerousness and uncontrollability are not simultaneously addressed, increases in warmth alone may be insufficient to reduce social distance. This theoretical tension supports the rationale for testing two distinct humanizing conditions that each target different components of this bidimensional stigma profile. In the present study, two such humanizing interventions were implemented: one with an empathy focus and another with a prevention focus.

The empathy-focused humanizing intervention (IN2) was designed to target narrative perspective-taking. Such personal narratives, presenting first-person accounts of individuals at risk, encourage audiences to imagine the internal experiences, emotions, and struggles of stigmatized individuals, thereby potentially encouraging perspective-taking and emotional engagement. Moreover, narrative exposure can simulate elements of intergroup contact [5], allowing audiences to experience empathy and perspective-taking even in the absence of direct interaction.

The persuasive effects of narrative messages depend critically on the degree to which audiences are cognitively and emotionally transported into the narrative world. Green and Brock [19] define *narrative transportation* as the absorption of attention, imagery, and affect into a story – the central mechanism underlying narrative persuasion. Transported readers show greater story-consistent belief change, are less likely to counterargue with the narrative's claims, and evaluate the story's protagonist more favorably; crucially, these effects are largely independent of whether the story is labeled as fact or fiction. Applied to the present study, the effectiveness of IN2 depends in part on whether participants achieve sufficient narrative engagement. In this study, the protagonist is portrayed as an individual coping with stigma and managing their attraction responsibly. The intervention was intended to emphasize emotional connection and perspective-taking as mechanisms to counteract dehumanization, primarily targeting the warmth dimension of the SCM stigma profile described above.

IN3 functions as a hybrid of both persuasion routes: its narrative format engages the affective route through humanization and perspective-taking, while its emphasis on self-regulation, agency, and behavioral controllability simultaneously engages the informational-cognitive route by providing reasoning-based grounds for revising threat appraisals. In SCM terms, this dual mechanism means IN3 targets both the warmth dimension – through the shared humanization element – and the controllability-competence dimension of the stigma profile, addressing the perceived-threat component.

The proposed intervention mechanisms and associated outcome domains are derived from prior theoretical and empirical literature. However, the present study does not directly measure the mediating psychological processes underlying these pathways. The listed outcomes (see Table 1) therefore represent theoretically associated domains rather than direct tests of mediation mechanisms.

**Table 1:** Outcome domains theoretically associated with the proposed intervention mechanisms

Intervention	Persuasion route	Mechanism	Targeted outcome measures
IN1 – Educational	Informational- cognitive	Psychoeducation: correction of misinformation via central-route cognitive elaboration of evidence-based content. Provides accurate information about pedophilia, the distinction between sexual interest and offending, and the availability and effectiveness of treatment. Targets the belief that sexual interest inevitably leads to offending (controllability; [36]) and that individuals with such interests pose an unavoidable, unchangeable threat (dangerousness; [36])	Stigma: controllability, dangerousness Literacy: about therapy availability and effectiveness Behavioral: openness toward the topic; behavioral intention/self-reported behavior
IN2 – Humanizing, empathy-focused	Affective-narrative	Empathy arousal trough narrative transportation: first-person account invites the reader to adopt the protagonist’s point of view. The potential emotional identification may activate empathy by presenting the protagonist as a full human being, potentially counteracting the dehumanizing associations that underlie stigma. Via the Stereotype Content Model [12], a shift toward pity/warmth may reduce emotional rejection. Humanizing information can improve behavioral intentions toward stigmatized outgroups [5]	Stigma: fear, anger, pity, social distance Behavioral: openness toward the topic; behavioral intention/self-reported behavior
IN3 – Humanizing, prevention-focused	Hybrid (affective + cognitive)	Humanization + self-regulation: the narrative voice is oriented toward prevention and help-seeking rather than inner emotional experience. The protagonist sought professional help, maintained behavioral control, and has not offended. This models self-regulation and positions offending as preventable, targeting controllability and perceived dangerousness [12]	Stigma: controllability, dangerousness Literacy: about therapy availability and effectiveness Behavioral: openness toward the topic; behavioral intention/self-reported behavior

All three interventions are expected to affect overall affective state (valence, arousal) by virtue of engaging with emotionally charged content, regardless of route. This is a cross-cutting prediction not specific to any one mechanism

### The present study

As outlined above, public communication strategies targeting the prevention of CSA/M aim to achieve multiple interrelated goals: to increase knowledge, reduce stigma, diminish negative affective reactions, and promote openness and acceptance of support services among the general public. For individuals at risk, such strategies may contribute to social conditions that are theoretically relevant for help-seeking and prevention efforts. While several studies have emphasized the potential of psychoeducation, stigma reduction, and narrative-based messaging, empirical evidence regarding the effectiveness of these approaches remains limited to specific samples, e.g., English-only or student samples.

In particular, there is a lack of systematically evaluated, randomized, controlled studies that assess the impact of specific messages in quasi-representative general population samples, especially outside English-speaking contexts. Moreover, cross-cultural differences in public perception of pedophilia, CSAM, and rehabilitation suggest that messaging strategies may not be equally effective across countries or sociocultural settings [30]. Yet much of the current literature is based on studies from North America, the UK, and Australia, leaving a gap in understanding how such interventions work in other cultural contexts.

The present study aims to address this gap by systematically testing the three different intervention messages in a randomized controlled online experiment conducted with a quasi-representative sample from two non-English-speaking countries. To translate the outlined challenges into concrete intervention strategies, the present study tests communication approaches that were developed drawing on mechanisms of stigma reduction, attitude change, and behavior change techniques. Specifically, we investigated whether exposure to educational and humanizing interventions featuring an intervention for individuals at risk, in comparison to a control condition, had a measurable impact on outcomes related to knowledge, affective reactions, and stigmatization. The educational intervention sought to address knowledge gaps and misinformation. This type of messaging is grounded in the idea that accurate information can reduce stigmatizing attitudes by correcting misconceptions. A humanizing intervention with an empathy focus was designed to foster emotional perspective-taking and thereby reducing dehumanization and stigma. A humanizing narrative with a prevention focus aimed to enhance perceived behavioral control, personal agency, and belief in changeability, which are critical components for both public support of rehabilitative policies and for motivating at-risk individuals to seek help.

Given the limited prior experimental research on brief stigma-reduction and prevention-oriented communication interventions in the context of CSA/M, the present study was designed as an exploratory investigation. Accordingly, all analyses were considered exploratory and hypothesis-generating rather than confirmatory tests. The primary research questions (RQ) were as follows:

- RQ1a: Do educational vs. humanizing with prevention focus vs. humanizing with empathy focus interventions have an effect on attitude, behavioral intention, cognition and affect compared to a control group message?
- RQ1b: Do the interventions differ in their effectiveness with respect to the outcomes attitude, behavioral intention, cognition and affect?
- RQ1c: Are there any adverse effects, such as negative affective responses or increased stigma, that raise ethical concerns regarding message content or framing?

In addition, as secondary research questions, we explored:

- RQ2a: Does the intervention impact differ as a function of participants’ demographics (e.g., having children, cultural background)?
- RQ2b: Does the type of intervention have an impact on the dropout rate?

## Methods

The study was retrospectively registered at German Clinical Trials Register (DRKS) (DRKS00038927, 09/01/2026). The registration was based on the study aims, procedures, and analytic plans as specified in the original ethics application submitted in July 2023 and approved in August 2023, prior to data collection. Given the exploratory nature of the study and the limited prior experimental literature on brief stigma-reduction interventions in the context of CSA/M prevention communication, all analyses were treated as exploratory and hypothesis-generating rather than confirmatory tests.

### Ethics

The study protocol, including all intervention materials and procedures, was reviewed and approved by the responsible ethics committee. The study was classified as involving minimal risk. Prior to participation, all participants completed an informed consent procedure. Participants were informed that the survey would include questions related to sexual experiences, about the voluntary nature of participation and the possibility to discontinue participation at any time. Participants were provided with information about available support resources for individuals affected by sexual violence. The study was registered based on the ethics application. However, several analytic refinements were developed post hoc since adjustments were necessary due to the data structure.

### Participants

The participants were recruited via an agency (IntraResearch). The recruitment started in September 2023; the initial survey took place in mid-September 2023 for the Spanish sample and in early October 2023 for the Portuguese sample. Potential participants were invited to take part in a survey about a health information campaign and were provided with detailed study information. Participants received a standard compensation for their participation.

To be included in the study, participants had to be at least 18 years old, live in Portugal or Spain, and had to have sufficient language skills in Spanish or Portuguese. The average survey completion time for the initial survey was *M* = 10.60 min (*SD* = 3.93), and for the follow-up survey *M* = 5.01 min (*SD* = 2.61). As the present study was designed as a public-attitude study in a general population sample rather than a study targeting individuals at risk themselves, participants reporting a sexual interest in children or prior CSAM consumption were excluded from the sample at hand. Exclusion criteria were assessed at the end of the survey procedure in order to minimize drop-outs.

**Fig. 1:**
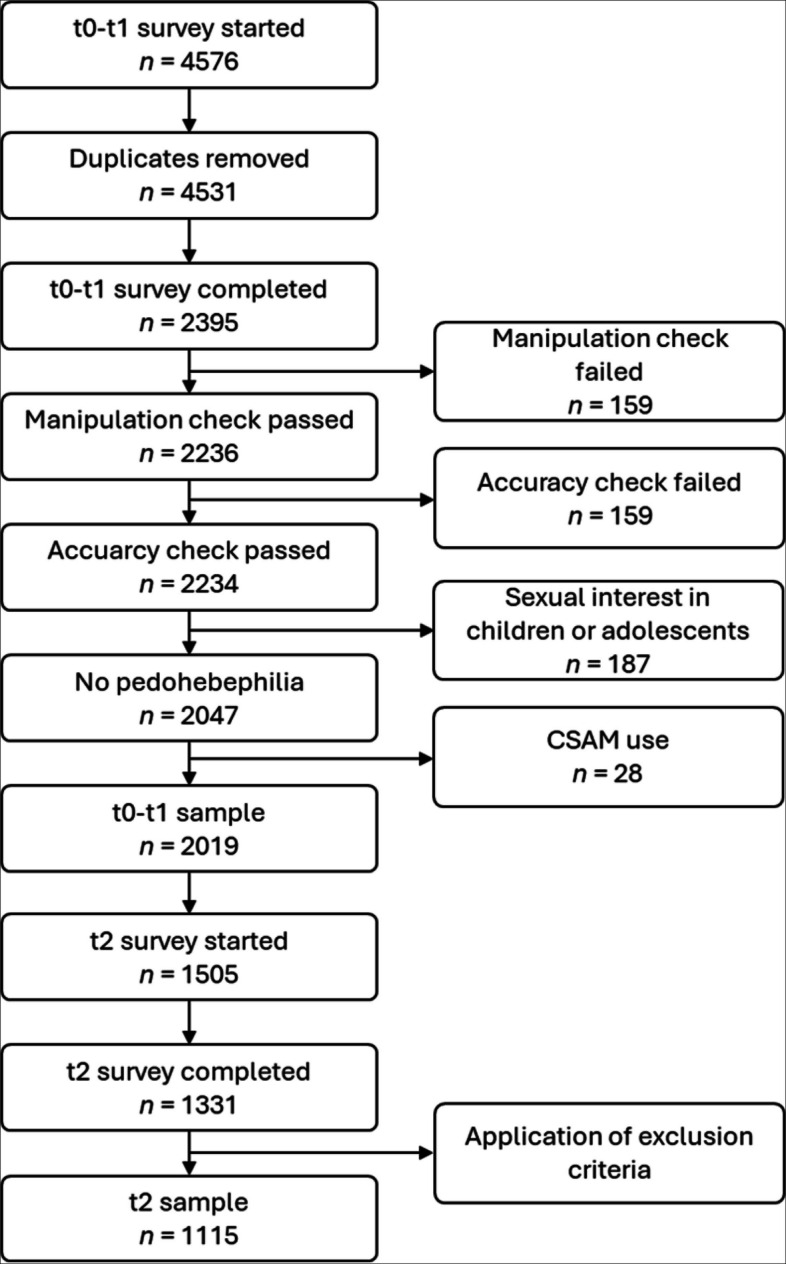
Participant inclusion and exclusion flowchart

The initial sample consisted of *N* = 2395 participants who completed the first survey. Of these, *n* = 376 participants were excluded for different reasons (poor data quality,^1^ multiple responses, sexual preference, CSAM consumption), resulting in a final sample size of *N* = 2019 for the initial survey. *n* = 1331 participants completed the follow-up survey; *n* = 1115 were found to be valid for the follow-up survey which corresponds to a return rate of 55.23% (Spanish: 46.65%; Portuguese: 64.17%). Figure 1 outlines in exclusion process leading to the final sample in detail. The demographic characteristics of the initial sample are depicted in Table 2.

**Table 2:** Participant demographics (initial survey)

	**Language**	
	**Portuguese**	**Spanish**
Mean age (SD)	44.15 (12.84)	43.55 (12.49)
Gender		
female (%)	489 (49.49)	557 (54.03)
male (%)	499 (50.51)	474 (45.97)
Living environment		
large city (%)	335 (33.91)	535 (51.89)
medium-sized city (%)	424 (42.91)	300 (29.10)
small city (%)	229 (23.18)	196 (19.01)
Children (%)	626 (63.36)	656 (63.63)
Education		
none (%)	1 (0.10)	1 (0.10)
school (%)	50 (5.06)	118 (11.45)
any kind of diploma that proves general qualification for university entrance (e.g., high school diploma) (%)	253 (25.61)	142 (13.77)
vocational education (%)	120 (12.15)	288 (27.93)
Bachelor’s or equivalent level (%)	381 (38.56)	336 (32.59)
Master’s or equivalent level (%)	164 (16.60)	118 (11.45)
doctoral or equivalent level (%)	19 (1.92)	28 (2.72)

### Procedure

**Fig. 2:**
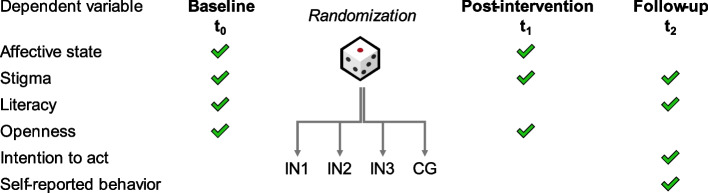
Study design

Informed consent was obtained from all participants prior to their inclusion in the study. Data were collected via online questionnaires hosted on SoSci Survey. The study included two surveys: the baseline measurement (t_0_) and the post-intervention measurement (t_1_) were realized in one survey. A follow-up survey (t_2_) was administered 3 to 4 weeks after the initial survey. Figure 2 illustrates the study design.

Following the baseline assessment (t_0_), participants were randomly assigned to the experimental group via SoSci Survey using a built-in randomizer. No stratification was applied. Participants were unaware of the study hypotheses and were blind to the group allocation. The intervention aimed to examine the effects of different health communication strategies on knowledge, stigma, affective response, and behavioral intentions in the context of CSAM prevention. Each participant received one of the following message formats (the messages are provided in Table 3):

- Intervention 1 (IN1) – educational message: Participants in this group were presented with a short, text-based informational message providing accurate and destigmatizing facts about CSAM, pedophilia, and treatment effects [22, 32]. The term “so-called ‘child pornography’” was chosen because it is more familiar to the general population than “child sexual abuse material”. We are aware that this does not correspond to the recommended terminology explained at the beginning. To emphasize that the term must be questioned, “so-called” has been added each time the term is mentioned. The message clarified the distinction between pedophilia and offending behavior, emphasized the criminal nature and consequences of CSAM consumption, and highlighted the availability and effects of therapeutic support for individuals at risk.
- Intervention 2 (IN2) – humanization with empathy focus: This condition featured a personal quote from “Luis,” a therapy client who described the emotional challenge and relief of disclosing his attraction and seeking help. The quote is deliberately vague so that it could refer to sexual attraction to children as well as the use of CSAM. The narrative was designed to foster empathy and identification by providing a realistic and relatable role model [1, 22].
- Intervention 3 (IN3) – humanization with prevention focus: In this condition, participants read a quote from “Luis” expressing his motivation to avoid offending behavior and his engagement in therapy as a preventive strategy. This message emphasized self-control, treatment availability and efficacy, and personal responsibility, aiming to increase belief in changeability and to reduce punitive responses [32, 50].
- Control group (CG): Participants in the control group received no CSAM or pedophilia related content but general information about a rather common disease in accordance with the framing of a health information campaign.

**Table 3:** Interventions and control group message

Group	Video clip	Text
IN1	Man watching CSAM who is interrupted by his partner. He abruptly closes his laptop. At the end of the video the text “There is help – www.troubled-desire.com” is displayed. The video is accompanied by thematically appropriate background sound. (duration: 18 s)	Facts about so-called “child pornography”: • The production, distribution and use of images or videos depicting child sexual abuse (so-called “child pornography”) is a serious criminal offense to the detriment of children • The use of child sexual abuse materials or the desire to do so may indicate pedophilia • Pedophilia ≠ Pedophilic disorder: Not every person with pedophilia becomes an offender, and not every offender is a pedophile • Therapy can help individuals who use child sexual abuse materials or fear to do so to control their behavior while improving life satisfaction
IN2		Luis, 28: “Talking about it for the first time wasn't easy, especially since I'm not one to talk about my feelings in a big way. It's quite difficult, but it's also a relief, because I've been carrying that around with me for years. And then there's someone who doesn't immediately say, you're the last scum of the earth, who also knows about the whole topic, which is also important.”
IN3		Luis, 28: “I don't even want to be tempted to look at child pornography. Therapy is supposed to help me lead a life without ever committing a sexual assault on a child, and it supports me pretty well in that.”
CG	Person typing on a mobile phone with unspecific background music. At the end of the video the text “get advice” is displayed. (duration: 17 s)	Around 10.5% of the world's adult population suffer from the metabolic disease diabetes. If left untreated, diabetes can lead to a person's death, either directly or indirectly through concomitant diseases. A healthy body weight, regular exercise and a balanced diet can reduce the risk of developing diabetes

Each intervention group was exposed to the same video clip, accompanied by one of three different texts (see Table 3). This design choice was intentional and aimed to ensure that comparisons between intervention conditions reflected differences in message framing rather than differences in topic exposure or audiovisual salience. Holding the thematic stimulus constant allowed the study to isolate, as far as possible, the differential effects of educational, empathy-focused, and prevention-focused communication strategies under comparable contextual conditions.

The control group received a text about diabetes and a neutral, unrelated video. The control condition was designed to provide a neutral health-communication comparison stimulus unrelated to CSA/M. However, because the control condition differed from the intervention conditions not only in message framing but also in thematic and emotional salience, it does not constitute a topic-matched control condition.

The interventions and the control stimuli were displayed for at least 30 s before participants were able to proceed to the next page of the survey. Participants spent an average of 55.87 s on the intervention page (SD = 93.68).^2^ Each intervention message was combined with references to help offers for people who use CSAM (“Help for people who use child sexual abuse material (so-called ‘child pornography’): [you will find the original link at the end of the survey]”) and help offers for victims of sexual abuse (“Help for victims of sexual violence: [you will find the original link at the end of the survey]”). The interventions as well as the control group message were applied in Spanish and Portuguese. Therefore, we chose testimonial names that are common in both languages. The interventions were intentionally designed as brief, single-exposure communication stimuli. Brief and isolated exposures reflect realistic patterns of contemporary digital media consumption, in which individuals frequently encounter short-form public communication messages in fragmented and transient formats (e.g., social media posts, short videos, or brief awareness campaigns).

### Measures

The outcome measures were selected to reflect the primary psychological and behavioral targets, as discussed above, of public health communication campaigns addressing CSAM prevention.

### Affective state

The affective state of participants was measured using the Affect Grid [47]. The Affect Grid is a single item scale that assesses the current affect along the dimensions of “pleasure – displeasure” and “arousal – sleepiness”. In the survey tool, the two dimensions were each scaled from 0 to 361, whereby a higher value on the dimension valence indicated a positive state and on the dimension arousal a state of low arousal. The original items were translated into Spanish and Portuguese, reviewed by native speakers and translated back to English to assure the appropriateness of translations. Participants assessed their affective state immediately before (t_0_) and after the intervention (t_1_).

### Stigma

Stigmatizing attitudes towards people with a dominant sexual interest in children were determined using the stigma scale by Jahnke et al. [27, 28]. The participants completed the stigma scale at all three measurement time points. The scale consists of four subscales, namely controllability, dangerousness, affective reaction, and social distance. Items were rated on 7-point Likert scales (1 = “do not agree at all” to 7 = “completely agree”).

The social distance subscale (6 items) was disaggregated into two components following Jahnke [26], who explicitly removed the two punitive attitude items from the scale “to achieve a ‘purer’ measure of social distance as opposed to punitive attitudes” (p. 366). Accordingly, *social avoidance* was computed as the mean of the four avoidance-oriented items (“would have these persons as friends,” “would accept these persons in my neighborhood,” “would accept these persons as colleagues at work,” “would talk to them”; items reverse-coded so that higher scores indicate greater avoidance). *Punitive attitudes* was computed as the mean of the two punitive items (“should be incarcerated,” “should better be dead”; higher scores indicate stronger punitive orientation). This disaggregation allows separate examination of behavioral avoidance and punitive response tendencies as theoretically distinct components of stigma.

In accordance with the authors’ instructions, the items of the subscale “controllability” were summarized to form an average score. The items “dangerousness for children” and “dangerousness for adolescents” were also combined into one score. All other items were considered individually in the evaluation. The reverse coded items (social avoidance items) were re-coded so that higher scores correspond to higher stigma values. The original items were translated into Spanish and Portuguese, reviewed by native speakers and translated back to English to assure the appropriateness of translations. The internal consistency of the scales in the current sample was excellent for “avoidance/social distance” (Cronbach’s a = .92) and good for “controllability” (Cronbach’s a = .84) and “dangerousness” (Cronbach’s Alpha = .81, *r* = .67). For the “punitive response” subscale was internal consistency limited, with a Cronbach's a of .64 and an inter-item correlation of *r* = .47.

### Literacy

Participants were asked to rate their knowledge about the availability of therapeutic offers for people who use CSAM and the perceived effectiveness of therapeutic offers on a 5-point Likert scale (1 = “not at all”, 5 = “very well”). The literacy-related questions were gathered before the intervention (t_0_) and in the follow-up survey (t_2_).

### Openness, intention to act and self-reported behavior

As a behavioral correlate of openness to the topic of CSAM and pedophilia, participants were asked to rate the extent to which they were interested in learning about the topic and would visit a relevant website (t_0_, t_1_). Those two variables were integrated into the “openness” scale by averaging responses of both items (Cronbach’s Alpha = 0.924, *r* = 0.859). In the follow-up survey (t_2_), participants were asked if they would visit the website “Troubled Desire” and if they had visited the website since participating in the study. All questions, with the exception of the question about the actual website visit, were answered on a 5-point Likert scale (1 = “not at all”, 5 = “very much”).

### Analyses

Descriptive statistics were calculated for all outcome variables to describe the sample and for an initial exploration of potential intervention effects. Mean, standard deviation, median and absolute number of cases were recorded for each dependent variable separately according to time of data collection (t0, t1, t2) and experimental condition (IN1, IN2, IN3, CG). Absolute and relative frequencies were calculated for categorical data. Additionally, we examined response distributions by calculating skewness, kurtosis, ceiling and floor effects, where applicable. No procedures were used to fill in missing data, as this was not necessary due to the survey configuration.

To address RQ1 (“Do the interventions impact stigma, affective states, and help-seeking behaviors?”), linear mixed-effects models (LMM) served as the primary inferential framework across all continuous outcome domains. LMMs were fitted with fixed effects for intervention group, time, and their interaction, with random intercepts for participants to account for repeated-measures dependency. This approach is robust to moderate violations of the normality assumption at the sample sizes obtained in the present study (N ˜ 2,000) by the Central Limit Theorem [37]. All primary analyses compared the three intervention groups to the control group. For outcome-specific features, such as ordinal measurement with three timepoints, severe ceiling effects, or categorical response formats, supplementary analytical methods were employed as described below.

- Affective state (valence and arousal): LMMs with heteroscedastic variance structures were fitted using the *nlme* package. Models included fixed effects for intervention group, time, and their interaction, with random intercepts for participants. Heteroscedastic variance structures were applied where Levene's tests indicated unequal variances across groups and timepoints. Parameter estimates with 95% confidence intervals were extracted to quantify effect directions and magnitudes.
- Aggregated stigma dimensions (controllability, social distance/avoidance, punitive attitudes, dangerousness for children and adolescents): For the t0–t1 interval, LMMs were fitted as the primary analysis consistent with the unified framework above. Across all three timepoints (baseline, post-intervention, follow-up), nonparametric longitudinal data analysis (nparLD; [40]) was additionally conducted as a robustness and sensitivity analysis, appropriate given the ordinal measurement scale and potential distributional violations. Models tested group, time, and group × time interaction effects using ANOVA-type statistics (ATS) with Satterthwaite degrees-of-freedom corrections. To quantify the magnitude of pairwise time-interval comparisons, rank-biserial correlation (*r*) was computed as the effect size [10]. This effect size is distribution-free, directly interpretable as the probability that a randomly selected observation from one timepoint exceeds one from another, and is the appropriate paired effect size metric for nonparametric longitudinal analyses [52]. Effect sizes were interpreted using the benchmarks: negligible (< .10), small (.10), medium (.30), and large (.50).
- Single-item stigma measures (adult dangerousness, affective reactions anger, fear, pity): These items exhibited severe ceiling effects and bimodal distributions that precluded stable LMM or nparLD estimation. Nonparametric cross-sectional comparisons (Kruskal–Wallis tests) were therefore conducted at post-intervention timepoints as the most appropriate alternative, given that distributional assumptions could not be met regardless of sample size. Effect sizes were quantified using epsilon-squared (ε²) and interpreted according to established benchmarks.
- Literacy, openness and behavioral intentions: For openness, LMMs were fitted with group × time interactions across two timepoints (baseline, post-intervention). The large sample size (*N* = 2,019) provides sufficient robustness against normality violations via the Central Limit Theorem [37]. For literacy, cumulative link mixed models (CLMM) with logit links were initially considered for the 5-point Likert scale assessed at two timepoints (baseline, follow-up); however, the proportional odds assumption was not adequately met, and LMMs were retained as the primary approach with robustness justification as above. For information-seeking intentions, a one-way ANOVA was conducted on cross-sectional follow-up data. For actual self-reported behavior, chi-square tests and logistic regression were used to analyze binary self-reported website visits at follow-up.

RQ1b (“Do the interventions differ in their effectiveness with respect to the outcomes attitude, behavioral intention, cognition and affect?”) was addressed using post-hoc comparisons. When omnibus tests indicated potential group differences (*p* < .10), pairwise comparisons were conducted using method-appropriate approaches: Tukey HSD with family-wise error rate control for parametric outcomes; Dunn's test with Bonferroni correction for nonparametric outcomes; custom contrasts within the ordinal mixed-effects framework with Bonferroni adjustment for ordinal outcomes; pairwise chi-square tests with Bonferroni correction for categorical outcomes.

Potential adverse effects (RQ1c) were assessed by systematically examining parameter estimates and effect directions to identify patterns suggesting increased stigma, reduced help-seeking, or other negative outcomes. This included inspection of confidence intervals for parameter estimates, effect size magnitudes, and patterns of change across timepoints. For LMM outcomes, partial eta-squared (η^2^p) was used as the primary effect size metric, interpreted using conventional benchmarks: small (0.01), medium (0.06), and large (0.14) for partial eta-squared [8]. Effects below .01 were considered negligible regardless of *p*-values [11].

To address RQ2a (“Does the intervention impact differ as a function of participants' demographics?”), moderation analyses were conducted examining whether demographic characteristics influenced intervention effectiveness. All moderation analyses were conducted as exploratory post-hoc investigations and are interpreted as hypothesis-generating; given their explicitly exploratory status, no multiplicity correction was applied within the moderation analyses themselves. Demographic moderators examined were: age (mean-centered), gender, city size, parental status (having children), education level, adverse childhood experiences (ACE), and primary language. For continuous outcomes, LMMs with heteroscedastic variance structures were fitted using the nlme package, including fixed effects for intervention group, time, demographic variables, and all relevant two-way and three-way interactions, with random intercepts for participants. Parameter estimates with 95% confidence intervals were extracted for significant interaction effects to quantify moderation magnitude and direction. For ordinal stigma outcomes across three timepoints, nparLD models tested intervention × time × demographic interactions using F-statistics, with separate analyses conducted for controllability, social distance, and dangerousness. For literacy, CLMMs with logit links tested intervention × time × demographic interactions via likelihood ratio tests comparing nested models. For the omnibus test of moderation across all outcomes and demographics, Benjamini–Hochberg false discovery rate (FDR) correction was applied within each outcome domain to control Type I error inflation while maintaining reasonable statistical power.

For RQ2b (“Does the type of intervention have an impact on the dropout rate?”), study attrition was analyzed by intervention group. Dropout was defined as not providing data at t2 (follow-up). First, we tested whether dropout rates differed across the four intervention conditions using a chi-square test of independence. Second, completers and dropouts were compared on all baseline (t0) measures, including demographic, psychological and behavioral variables. Continuous variables were compared using Welch's t-tests with Cohen's d as effect size; categorical variables were compared using chi-square or Fisher's exact tests with Cramér's V. All *p*-values were corrected for multiple testing using the Benjamini–Hochberg FDR procedure across the full set of comparisons (k = 19).

All analyses were conducted in R (v4.3.1; [45]). LMMs were fitted with *lme4* [3] and *nlme* [41]; CLMMs with *ordinal* [6]; nonparametric longitudinal models with *nparLD* [40]. R base functions were used for ANOVA and chi-square tests. Estimated marginal means and contrasts were obtained via *emmeans* [34]; effect sizes via *effectsize* [4]. Model diagnostics and assumption checks used *car* [14] and base stats; data handling and visualization used the *tidyverse* [54] and *ggplot2* [53]. To support the development of data analysis scripts, AI-based code generation tools (GitHub Copilot/Claude) were used to assist in writing and debugging R code for statistical analyses and data visualization. All automatically generated code was reviewed by the authors to ensure accuracy and validity.

## Results

In the following, the descriptive results are first reported and then the research questions posed at the beginning are addressed. All descriptive values can be found in Table 4.

**Table 4:** Descriptive statistics of all outcome variables

Outcome	Group	Timepoint	*N*	*M*	*SD*	CI	Median
Affective state – valence	IN1	t0	500	181.93	103.40	173, 191	183.0
		t1	500	146.47	101.54	138, 155	144.5
	IN2	t0	498	179.60	105.75	170, 189	183.0
		t1	498	145.10	104.29	136, 154	142.5
	IN3	t0	503	180.40	103.56	171, 189	183.0
		t1	503	149.64	98.42	141, 158	148.0
	CG	t0	518	184.99	107.95	176, 194	186.0
		t1	518	198.30	97.52	190, 207	189.0
Affective state – arousal	IN1	t0	500	171.79	97.51	163, 181	180.0
		t1	500	177.46	78.31	170, 184	181.0
	IN2	t0	498	174.88	98.50	166, 184	180.5
		t1	498	178.19	76.95	171, 185	181.0
	IN3	t0	503	181.22	95.61	173, 189	181.0
		t1	503	182.35	81.21	175, 189	182.0
	CG	t0	518	179.03	101.00	170, 188	180.5
		t1	518	197.37	92.81	189, 205	186.0
Stigma – controllability	IN1	t0	500	4.04	2.06	3.86, 4.22	4.00
		t1	500	4.11	2.06	3.93, 4.29	4.00
		t2	282	3.89	2.11	3.64, 4.14	3.67
	IN2	t0	498	4.18	2.09	4.00, 4.37	4.00
		t1	498	4.15	2.21	3.96, 4.35	4.00
		t2	258	4.10	2.04	3.85, 4.35	4.17
	IN3	t0	503	4.20	2.08	4.02, 4.39	4.00
		t1	503	4.19	2.16	4.00, 4.38	4.00
		t2	285	3.98	1.98	3.75, 4.21	4.00
	CG	t0	518	3.92	2.02	3.74, 4.09	4.00
		t1	518	3.97	2.10	3.79, 4.16	4.00
		t2	290	3.99	2.10	3.75, 4.23	4.00
Stigma – dangerousness for children and adolescents	IN1	t0	500	6.58	1.00	6.49, 6,66	7.00
		t1	500	6.39	1.19	6.29, 6.50	7.00
		t2	282	6.33	1.31	6.18, 6.48	7.00
	IN2	t0	498	6.56	1.03	6.47, 6.65	7.00
		t1	498	6.38	1.24	6.28, 6.49	7.00
		t2	258	6.32	1.26	6.16, 6.47	7.00
	IN3	t0	503	6.57	1.06	6.48, 6.66	7.00
		t1	503	6.50	1.15	6.40, 6.60	7.00
		t2	285	6.40	1.14	6.27, 6.53	7.00
	CG	t0	518	6.48	1.15	6.38, 6.58	7.00
		t1	518	6.45	1.15	6.35, 6.55	7.00
		t2	290	6.38	1.16	6.25, 6.52	7.00
Stigma – dangerousness for adults	IN1	t0	500	4.98	2.30	4.78, 5.18	6.00
		t1	500	4.89	2.30	4.69, 5.10	6.00
		t2	282	4.67	2.37	4.40, 4.95	5.00
	IN2	t0	498	5.03	2.28	4.83, 5.23	6.00
		t1	498	4.93	2.30	4.73, 5.13	6.00
		t2	258	4.72	2.34	4.43, 5.00	5.00
	IN3	t0	503	5.11	2.28	4.91, 5.31	6.00
		t1	503	5.02	2.30	4.82, 5.22	6.00
		t2	285	4.62	2.32	4.36, 4.89	5.00
	CG	t0	518	4.80	2.32	4.60, 5.00	6.00
		t1	518	4.79	2.31	4.60, 5.00	5.00
		t2	290	4.70	2.63	4.43, 4.98	5.00
Stigma – affective reaction: fear	IN1	t0	500	5.64	1.99	5.47, 5.81	7.00
		t1	500	5.31	2.17	5.12, 5.50	7.00
		t2	282	5.31	2.13	5.06, 5.56	6.00
	IN2	t0	498	5.67	1.98	5.50, 5.85	7.00
		t1	498	5.43	2.08	5.24, 5.61	7.00
		t2	258	5.43	2.03	5.19, 5.68	6.00
	IN3	t0	503	5.70	2.05	5.52, 5.88	7.00
		t1	503	5.45	2.14	5.26, 5.63	7.00
		t2	285	5.36	2.12	5.22, 5.63	7.00
	CG	t0	518	5.50	2.09	5.32, 5.68	7.00
		t1	518	5.30	2.17	5.12, 5.49	6.50
		t2	290	5.27	2.18	5.02, 5.52	6.00
Stigma – affective reaction: pity	IN1	t0	500	3.34	2.46	3.12, 3.55	2.00
		t1	500	3.70	2.43	3.50, 3.91	4.00
		t2	282	3.54	2.44	3.26, 3.83	3.00
	IN2	t0	498	3.16	2.37	2.95, 3.36	2.00
		t1	498	3.51	2.38	3.31, 3.72	3.00
		t2	258	3.47	2.36	3.18, 3.75	3.00
	IN3	t0	503	3.05	2.42	2.84, 3.26	2.00
		t1	503	3.42	2.46	3.24, 3.63	3.00
		t2	285	3.16	2.31	2.89, 3.43	2.00
	CG	t0	518	3.40	2.46	3.16, 3.61	3.00
		t1	518	3.54	2.44	3.33, 3.75	3.00
		t2	290	3.40	2.36	3.12, 3.67	3.00
Stigma – affective reaction: anger	IN1	t0	500	6.27	1.44	6.15, 6.40	7.00
		t1	500	6.02	1.65	5.87, 6.16	7.00
		t2	282	6.06	1.66	5.87, 6.26	7.00
	IN2	t0	498	6.17	1.55	6.03, 6.30	7.00
		t1	498	5.96	1.69	5.81, 6.10	7.00
		t2	258	5.92	1.76	5.71, 6.14	7.00
	IN3	t0	503	6.37	1.41	6.25, 6.49	7.00
		t1	503	6.17	1.56	6.04, 6.31	7.00
		t2	285	6.13	1.55	5.94, 6.31	7.00
	CG	t0	518	6.22	1.44	6.10, 6.35	7.00
		t1	518	5.97	1.77	5.81, 6.12	7.00
		t2	290	6.03	1.56	5.85, 6.21	7.00
Stigma – avoidance/social distance^1^	IN1	t0	496	5.97	1.21	5.86, 6.07	6.50
		t1	496	5.89	1.25	5.78, 6.00	6.25
		t2	282	5.73	1.30	5.59, 5.90	6.00
	IN2	t0	496	5.91	1.22	5.80, 6.03	6.33
		t1	495	5.80	1.28	5.69, 5.91	6.25
		t2	257	5.62	1.29	5.46, 5.78	5.75
	IN3	t0	500	6.11	1.14	6.01, 6.21	6.75
		t1	497	6.00	1.23	5.88, 6.10	6.50
		t2	285	5.81	1.28	5.66, 5.96	6.25
	CG	t0	513	5.95	1.20	5.84, 6.05	6.50
		t1	514	5.94	1.22	5.83, 6.04	6.25
		t2	286	5.83	1.20	5.69, 5.97	6.00
Stigma – punitive responses^1^	IN1	t0	442	3.08	1.43	2.95, 3.21	3.00
		t1	447	2.80	1.44	2.66, 2.93	2.50
		t2	253	2.98	1.37	2.81, 3.15	3.00
	IN2	t0	432	2.87	1.41	2.73, 2.30	3.00
		t1	451	2.57	1.45	2.44, 2.71	2.50
		t2	226	2.79	1.37	2.61, 2.97	3.00
	IN3	t0	429	2.99	1.48	2.85, 3.14	3.00
		t1	443	2.65	1.50	2.51, 2.79	2.50
		t2	250	2.85	1.40	2.67, 3.02	2.50
	CG	t0	464	2.81	1.40	2.68, 2.94	2.75
		t1	473	2.55	1.40	2.42, 2.68	2.50
		t2	261	2.86	1.45	2.69, 3.04	3.00
Literacy – therapy availability	IN1	t0	500	2.27	1.23	2.16, 2.38	2.00
		t2	282	2.31	1.26	2.17, 2.47	2.00
	IN2	t0	498	2.09	1.16	1.99, 2.19	2.00
		t2	258	2.32	1.20	2.18, 2.47	2.00
	IN3	t0	503	2.29	1.22	2.18, 2.39	2.00
		t2	285	2.31	1.16	2.17, 2.44	2.00
	CG	t0	518	2.24	1.22	2.13, 2.35	2.00
		t2	290	2.32	1.24	2.18, 2.47	2.00
Literacy – therapy effectiveness	IN1	t0	500	3.33	1.21	3.23, 3.44	3.00
		t2	282	3.28	1.21	3.14, 3.42	3.00
	IN2	t0	498	3.34	1.20	3.24, 3.45	3.00
		t2	258	3.26	1.17	3.12, 3.40	3.00
	IN3	t0	503	3.40	1.19	3.30, 3.50	3.00
		t2	285	3.24	1.13	3.11, 3.37	3.00
	CG	t0	518	3.31	1.18	3.20, 3.41	3.00
		t2	290	3.27	1.21	3.13, 3.41	3.00
Openness	IN1	t0	500	2.80	1.23	2.77, 2.99	3.00
		t1	500	2.68	1.22	2.59, 2.81	3.00
	IN2	t0	498	2.73	1.24	2.68, 2.90	3.00
		t1	498	2.69	1.21	2.58, 2.79	3.00
	IN3	t0	503	2.86	1.22	2.61, 2.84	3.00
		t1	503	2.65	1.24	2.49, 2.71	3.00
	CG	t0	518	2.88	1.25	2.76, 2.97	3.00
		t1	518	2.70	1.27	2.54, 2.76	3.00
Intention to act – visit “Troubled Desire”	IN1	t2	282	2.61	1.27	2.46, 2.76	3.00
	IN2	t2	258	2.65	1.25	2.50, 2.80	3.00
	IN3	t2	285	2.77	1.25	2.62, 2.92	3.00
	CG	t2	290	2.80	1.30	2.66, 2.95	3.00
Self-reported behavior – visited “Troubled Desire”	IN1	t2	282	23 (8.15%)			
	IN2	t2	258	14 (5.43%)			
	IN3	t2	285	22 (7.72%)			
	CG	t2	290	15 (5.17%)			

^1^N may differ from the other variables because, for technical reasons, only one response was enforced for this scale. It is possible that individual items were left unanswered

### Affective state

On average, affect related to valence decreased after the intervention across all groups (*M*_*t0*_ = 181.76; *M*_*t1*_ = 160.22), with this decrease being particularly visible in the intervention groups (e.g. in IN1, *M*_*t0*_ = 181.93 decreased to *M*_*t1*_ = 146.47). The control group, on the other hand, showed an increase in positive affect (*M*_*t1*_ = 198.30). The arousal level remained relatively stable overall in the intervention groups, while it decreased slightly in the control group (from *M*_*t0*_ = 179.03 to *M*_*t1*_ = 197.37; higher values indicate lower arousal). At the beginning of the survey (t_0_), the participants’ affective valence was on average in the neutral range; they felt neither particularly positive nor negative. The arousal value was also on average in the medium range, which indicates an affectively balanced state between arousal and relaxation. After receiving one of the three intervention messages (IN1-IN3), there was a slight shift in affective valence towards unpleasant emotions. This shift was similarly pronounced in all three experimental groups. In the control group, on the other hand, there was a slight increase in positive affects (higher valence) with slightly lower arousal, which indicates a more relaxed and pleasant reaction to the neutral message.

### Stigma

At baseline (t_0_), participants exhibited relatively high levels of stigmatizing attitudes toward individuals with a sexual interest in children. On average, the controllability subscale indicated moderate endorsement of the belief that such individuals have control over their sexual preferences (*M*_*t0*_ = 4.08, *SD* = 2.07 on a 7-point scale). Perceived dangerousness was markedly high, especially in relation to children and adolescents (*M*_*t0*_ = 6.55, *SD* = 1.06), suggesting that respondents viewed individuals with such interests as posing a substantial risk. Perceived dangerousness toward adults was lower but still moderate to high (*M*_*t0*_ = 4.98, *SD* = 2.30). The affective reactions at baseline were also indicative of stigma: Participants reported strong feelings of fear (*M*_*t0*_ = 5.63, *SD* = 2.03) and anger (*M*_*t0*_ = 6.26, *SD* = 1.46) towards people with a sexual interest in children. In contrast, pity was rated considerably lower (*M*_*t0*_ = 3.24, *SD* = 2.43). Finally, the mean avoidance/social distance score (*M*_*t0*_ = 5.99, *SD* = 1.29) as well as punitive responses (*M*_*t0*_ = 2.94, *SD* = 1.31) at baseline reflected a tendency to maintain distance from individuals with a sexual interest in children across multiple social contexts.

Across all groups, stigma-related measures showed only small changes between baseline (t_0_), post-intervention (t_1_), and follow-up (t_2_). On the controllability dimension, there was no change from t_0_ (*M*_*t0*_ = 4.08, *SD* = 2.07) to t_1_ (*M*_*t1*_ = 4.11, *SD* = 2.14), followed by a slight decrease at t_2_ (*M*_*t2*_ = 3.99, *SD* = 2.06). Perceptions of dangerousness toward children and adolescents decreased slightly over time (*M*_*t0*_ = 6.55, *M*_*t1*_ = 6.43, *M*_*t2*_ = 6.36), while dangerousness toward adults also declined from baseline (*M*_*t0*_ = 4.98) to t_2_ (*M*_*t2*_ = 4.68). Affective responses mirrored these trends: Reported fear decreased steadily across all time points (*M*_*t0*_ = 5.63, *M*_*t1*_ = 5.37, *M*_*t2*_ = 5.34), and anger, while initially high (*M*_*t0*_ = 6.26), dropped slightly post-intervention and remained at that level at follow-up (t1 & t2: *M* = 6.03). In contrast, pity increased slightly from t_0_ (*M*_*t0*_ = 3.24) to t_1_ (*M*_*t1*_ = 3.54), with a minor decline at t_2_ (*M*_*t2*_ = 3.39). Finally, the avoidance/social distance score decreased modestly from t_0_ (*M*_*t0*_ = 5.99) to t_1_ (*M*_*t1*_ = 5.91) and remained nearly unchanged at t_2_ (*M*_*t2*_ = 5.75), indicating a small reduction in desire to socially exclude individuals with a sexual interest in children. Punitive responses scores decreased slightly from t_0_ (*M*_*t0*_ = 2.94) to t_1_ (*M*_*t1*_ = 2.64) and t_2_ (*M*_*t2*_ = 2.87).

### Literacy

At baseline (t_0_), participants’ literacy regarding the availability of therapeutic services for people who use CSAM was moderate across all groups, with an overall mean of 2.22 (SD = 1.21) on a 5-point scale. At follow-up (t_2_), a minimal overall increase was observed (M = 2.32, SD = 1.22). Beliefs about the effectiveness of therapy were moderately high at baseline (M = 3.35, SD = 1.00). At follow-up, the mean slightly decreased to 3.26 (SD = 1.18). This pattern was consistent across all groups.

### Openness, intention to act and self-reported behavior

At baseline (t_0_), participants reported a moderate openness to engaging with the topic of CSAM, with an overall mean of 2.82 (SD = 1.24) on a 5-point scale. This value decreased slightly at post-intervention (t_1_; M = 2.66, SD = 1.24), suggesting an overall drop in openness. When asked specifically about the likelihood of visiting the “Troubled Desire” website at follow-up (t_2_), participants across all conditions reported moderate interest (overall M = 2.71, SD = 1.28). Actual self-reported behavior was low overall: 74 participants (6.63%) indicated they had visited the “Troubled Desire” website since the study. The highest proportion of visits was reported in IN1 (8.13%) and IN3 (7.72%), followed by IN2 (5.43%) and the control group (5.17%).

#### RQ1a: Intervention effects compared to control

To examine whether the three intervention types (educational, humanizing with prevention focus, and humanizing with empathy focus) differed from the control condition in their effects on participants’ attitudes, behavioral intentions, cognitions, and affect, linear mixed-effects models, nonparametric Kruskal–Wallis tests, ANOVA, chi-square tests, and logistic regression models were applied as appropriate for the respective outcome domains. For valence, a significant group × time interaction emerged, *F*(3, 2015) = 32.750, *p* < .001, η^2^_p_ = .046, indicating a medium-sized effect. Participants’ affective responses varied over time depending on randomization group. For arousal, the group × time interaction was also significant, but small, *F*(3, 2015) = 4.142, *p* = .006, η^2^p = .006. Because higher arousal-grid scores indicate lower emotional activation, this pattern reflects only small group-dependent differences in changes in activation.

For stigma-related outcomes, no practically meaningful intervention effects were found for controllability, punitive responses, perceived dangerousness toward children/adolescents, dangerousness toward adults, fear, pity, or anger. Avoidance/social distance showed a statistically significant group × time interaction, *F*(6, 2735) = 2.942, *p* = .007, η^2^p = .006. However, the effect size was very small, suggesting limited practical relevance.

No significant group × time interactions were observed for openness toward the topic, perceived therapy availability, or perceived therapy effectiveness. Likewise, no significant group differences emerged for intention to visit the Troubled Desire website or for self-reported website visits. Detailed results are provided in Table 5.

**Table 5:** Outcomes per RQ

Outcome	Analytical approach	Results	Interpretation
*RQ1a: Intervention effects compared to control*			
Affective state – valence	LMM, homoscedastic (lmerTest); t0 + t1	Randomization group: F(3, 2015) = 11.856, *p* <.001, η^2^p =.023 Time: F(1, 2015) = 111.079, *p* <.001, η^2^p =.052 Group × time: F(3, 2015) = 32.750, *p* <.001, η^2^p =.046^1^	Significant group × time interaction (η^2^p =.046, medium)
Affective state – arousal	LMM, heteroscedastic (nlme); t0 + t1	Randomization group: F(3, 2015) = 0.927, *p* =.427, η^2^p =.001 Time: F(1, 2015) = 23.649, *p* <.001, η^2^p =.012 Group × time: F(3, 2015) = 4.142, *p* =.006, η^2^p =.006	Significant group × time interaction (η^2^p =.006, small)
Stigma – controllability	LMM, heteroscedastic (nlme); t0 + t1 + t2	Randomization group: F(3, 2015) = 2.159, *p* =.091, η^2^p =.003 Time: F(2, 3126) = 0.781, *p* =.458, η^2^p =.001 Group × time: F(6, 3126) = 1.357, *p* =.228, η^2^p =.003	No practical impact of the interventions on controllability scores relative to control
Stigma – avoidance/social distance	LMM, heteroscedastic (nlme); t0 + t1 + t2	Randomization group: F(3, 1965) = 3.738, *p* =.011, η^2^p =.006 Time: F(2, 2735) = 1.574, *p* =.207, η^2^p =.001 Group × time: F(6, 2735) = 2.942, *p* =.007, η^2^p =.006	Significant group × time interaction (η^2^p =.006, small)
Stigma – punitive responses	LMM, homoscedastic (lmerTest); t0 + t1 + t2	Randomization group: F(3, 1603.3) = 4.092, *p* =.007, η^2^p =.008 Time: F(2, 2042.8) = 55.838, *p* <.001, η^2^p =.052 Group × time: F(6, 2042.6) = 1.471, *p* =.184, η^2^p =.004	No significant group × time interaction, but time effect (η^2^p =.052)
Stigma – dangerousness for children and adolescents	LMM, heteroscedastic (nlme); t0 + t1 + t2	Randomization group: F(3, 2015) = 1.008, *p* =.388, η^2^p =.002 Time: F(2, 3126) = 1.368, *p* =.255, η^2^p =.001 Group × time: F(6, 3126) = 1.995, *p* =.063, η^2^p =.004	No practical impact of the interventions on dangerousness scores relative to control
Stigma – dangerousness for adults	Nonparametric tests (Kruskal–Wallis)	t1 (*N* = 2019): H(3) = 3.393, *p* =.335, e^2^ =.002 t2 (N = 1115): H(3) = 0.307, *p* =.959, e^2^ <.001	No differences were found
Stigma – affective reaction: fear	Nonparametric tests (Kruskal–Wallis)	t1 (*N* = 2019): H(3) = 2.500, *p* =.475, e^2^ =.001 t2 (*N* = 1115): H(3) = 0.351, *p* =.950, e^2^ <.001	No differences were found
Stigma – affective reaction: pity	Nonparametric tests (Kruskal–Wallis)	t1 (*N* = 2019): H(3) = 3.964, *p* =.265, e^2^ =.002 t2 (*N* = 1115): H(3) = 4.243, *p* =.236, e^2^ =.004	No differences were found
Stigma – affective reaction: anger	Nonparametric tests (Kruskal–Wallis)	t1 (*N* = 2019): H(3) = 5.815, *p* =.121, e^2^ =.003 t2 (*N* = 1115): H(3) = 1.819, *p* =.611, e^2^ =.002	No differences were found
Openness	LMM, homoscedastic (lmerTest); t0 + t1	Randomization group: F(3, 2015) = 1.060, *p* =.365, η^2^p =.002 Time: F(1, 2015) = 94.334, *p* <.001, η^2^p =.045 Group × time: F(3, 2015) = 2.165, *p* =.090, η^2^p =.003	All participants showed decreased openness to the topic from t_0_ to t_1_. No group differed from control in the magnitude of this change
Therapy availability	LMM, homoscedastic (lmerTest); t0 + t2	Randomization group: F(3, 2132.9) = 0.991, *p* =.396, η^2^p =.001 Time: F(1, 1369.5) = 7.133, *p* =.008, η^2^p =.005 Group × time: F(3, 1369.1) = 1.722, *p* =.161, η^2^p =.004	No significant group × time interaction. A general time effect (*p* =.008, η^2^p =.005, small) reflects a minimal overall increase in perceived therapy availability across all groups from t0 to t2
Therapy effectiveness	LMM, homoscedastic (lmerTest); t0 + t2	Randomization group: F(3, 2090.7) = 0.088, *p* =.967, η^2^p <.001 Time: F(1, 1463.9) = 5.383, *p* =.021, η^2^p =.004 Group × time: F(3, 1463.4) = 0.594, *p* =.619, η^2^p =.001	No differences were found
Intention to act – visit “Troubled Desire”	One-way ANOVA	*F*(3, 1111) = 1.43, *p* =.232, η^2^ =.004	No differences were found
Self-reported behavior – visited “Troubled Desire”	Chi-square test and logistic regression	^η2^(3) = 3.20, *p* =.361, Cramér's *V* =.054	No differences were found
*RQ1b: Differences between interventions*^2^			
Affective state – valence	Bonferroni pairwise contrasts (LMM EMMs at t1)	IN1 vs IN2: △ = 1.37, *p* = 1.000 IN1 vs IN3: △ = - 3.18, *p* = 1.000 IN2 vs IN3: △ = - 4.55, *p* = 1.000	No significant differences between intervention groups
Affective state – arousal	Bonferroni pairwise contrasts (LMM EMMs at t1)	IN1 vs IN2: △ = - 0.73, *p* = 1.000 IN1 vs IN3: △ = - 4.88, *p* = 1.000 IN2 vs IN3: △ = - 4.16, *p* = 1.000	No significant differences between intervention groups
Stigma – controllability	Bonferroni pairwise contrasts (LMM EMMs at t1 and t2)	All pairwise *p* >.05 (Bonferroni corrected)	No significant differences between intervention groups
Stigma – avoidance/social distance	Bonferroni pairwise contrasts (LMM EMMs at t1 and t2)	All pairwise *p* >.05 (Bonferroni corrected)	No significant differences between intervention groups
Stigma – punitive responses	Bonferroni pairwise contrasts (LMM EMMs at t1 and t2)	At t2: IN2 t1 vs IN1 t2 △ = - 0.411, *p* =.004; other pairwise ns	No significant pairwise differences at t1. One t1 → t2 cross-timepoint contrast significant (IN2 t1 > IN1 t2, *p* =.004), reflecting differential rebound of punitive responses at t2
Stigma – dangerousness for children and adolescents	Bonferroni pairwise contrasts (LMM EMMs at t1 and t2)	All pairwise *p* >.05 (Bonferroni corrected)	No significant differences between intervention groups
Openness	Bonferroni pairwise contrasts (LMM EMMs at t1)	All pairwise *p* >.05	No significant differences between intervention groups
Therapy availability	Bonferroni pairwise contrasts (LMM EMMs at t2)	All pairwise *p* >.05 (Bonferroni corrected)	No significant differences between intervention groups
Therapy effectiveness	Bonferroni pairwise contrasts (LMM EMMs at t2)	All pairwise *p* >.05 (Bonferroni corrected)	No significant differences between intervention groups
Self-reported behavior (visited “Troubled Desire”)	Pairwise chi-square tests with Bonferroni correction	All pairwise *p* >.05 (Bonferroni corrected)	No significant differences between intervention groups
*RQ1c: Potential adverse or ethical implications*			
Affective state – valence	LMM within-group estimates (△ t0 → t1)	CG: △ = + 13.3 [95% CI: + 5.3, + 21.3] IN1: △ = - 35.5 [95% CI: - 43.7, - 27.3] IN2: △ = - 34.5 [95% CI: - 42.7, - 26.3] IN3: △ = - 30.8 [95% CI: - 38.9, - 22.6]	After the intervention, the affective state on the valence dimension is more negative than before
Affective state – arousal	LMM within-group estimate (△ t0 → t1)	CG: △ = + 18.3 [95% CI: + 11.0, + 25.7] IN1: △ = + 5.7 [95% CI: - 1.8, + 13.2] IN2: △ = + 3.3 [95% CI: - 4.2, + 10.9] IN3: △ = + 1.1 [95% CI: - 6.4, + 8.6]	The control group showed an increase in arousal. Intervention groups showed non-significant increases
Stigma – controllability	LMM within-group estimate (△ t0 → t1)	CG: △ = + 0.057 [95% CI: - 0.062, + 0.176] IN1: △ = + 0.068 [95% CI: - 0.053, + 0.189] IN2: △ = - 0.026 [95% CI: - 0.148, + 0.096] IN3: △ = - 0.010 [95% CI: - 0.131, + 0.111]	Interventions have no effect of practical relevance
Stigma – avoidance/social distance	LMM within-group estimate (△ t0 → t1)	CG: △ = - 0.017 [95% CI: - 0.065, + 0.031] IN1: △ = + 0.081 [95% CI: + 0.032, + 0.130] IN2: △ = + 0.086 [95% CI: + 0.037, + 0.135] IN3: △ = + 0.103 [95% CI: + 0.055, + 0.151]	Although statistically significant reduction in avoidance (reverse coded items), practical relevance is negligible
Stigma – punitive responses	LMM within-group estimate (△ t0 → t1)	CG: △ = - 0.250 [95% CI: - 0.366, - 0.134] IN1: △ = - 0.337 [95% CI: - 0.454, - 0.220] IN2: △ = - 0.264 [95% CI: - 0.382, - 0.146] IN3: △ = - 0.417 [95% CI: - 0.541, - 0.293]	Although statistically significant reduction in punitive avoidance, practical relevance is negligible
Stigma – dangerousness for children and adolescents	LMM within-group estimate (△ t0 → t1)	CG: △ = - 0.024 [95% CI: - 0.107, + 0.059] IN1: △ = - 0.185 [95% CI: - 0.269, - 0.101] IN2: △ = - 0.178 [95% CI: - 0.263, - 0.093] IN3: △ = - 0.072 [95% CI: - 0.156, + 0.012]	Although statistically significant reduction in dangerousness in IN1 and IN2, practical relevance is negligible
Stigma – dangerousness for adults	Descriptive mean △ (t1 - t0)	CG: - 0.002 IN1: - 0.086 IN2: - 0.102 IN3: - 0.083	Interventions have no effect of practical relevance
Stigma – affective reaction: fear	Descriptive mean △ (t1 - t0)	CG: - 0.193 IN1: - 0.328 IN2: - 0.247 IN3: - 0.256	Interventions have no effect of practical relevance
Stigma – affective reaction: pity	Descriptive mean △ (t1 - t0)	CG: + 0.145 IN1: + 0.364 IN2: + 0.357 IN3: + 0.370	Interventions have no effect of practical relevance
Stigma – affective reaction: anger	Descriptive mean △ (t1 - t0)	CG: - 0.255 IN1: - 0.256 IN2: - 0.211 IN3: - 0.195	Interventions have no effect of practical relevance
Openness	LMM within-group estimate (△ t0 → t1)	CG: △ = - 0.181 [95% CI: - 0.244, - 0.118] IN1: △ = - 0.111 [95% CI: - 0.175, - 0.047] IN2: △ = - 0.127 [95% CI: - 0.191, - 0.063] IN3: △ = - 0.215 [95% CI: - 0.279, - 0.151]	Although statistically significant reduction in punitive avoidance, practical relevance is negligible
Therapy availability	LMM within-group estimate (△ t0 → t2)	CG: △ = + 0.070 [95% CI: - 0.053, + 0.193] IN1: △ = + 0.073 [95% CI: - 0.052, + 0.198] IN2: △ = + 0.202 [95% CI: + 0.073, + 0.331] IN3: △ = - 0.003 [95% CI: - 0.127, + 0.121]	Although IN2 showed a statistically significant increase in literacy about therapy availability, practical relevance is negligible
Therapy effectiveness	LMM within-group estimate (△ t0 → t2)	CG: △ = - 0.042 [95% CI: - 0.178, + 0.094] IN1: △ = - 0.054 [95% CI: - 0.192, + 0.084] IN2: △ = - 0.073 [95% CI: - 0.217, + 0.071] IN3: △ = - 0.161 [95% CI: - 0.299, - 0.023]	Although IN3 showed a statistically significant decrease in literacy about therapy effectiveness, practical relevance is negligible
Intention to act (visit “Troubled Desire”)	Welch one-way ANOVA (t2 only; cross-sectional)	F(3.0, 615.4) = 1.425, *p* =.234	Interventions have no effect of practical relevance
Self-reported behavior (visited “Troubled Desire”)	Logistic regression (t2 only)	IN1: OR = 0.614 [95% CI: 0.31, 1.20] IN2: OR = 0.951 [95% CI: 0.45, 2.01] IN3: OR = 0.652 [95% CI: 0.33, 1.28]	Interventions have no effect of practical relevance
*RQ2a: Moderating effects (exploratory)*			
Affective state – valence	LMM with 3-way Group × Time × Moderator interaction	No moderator significant after BH correction (all q >.05)	No demographic variable significantly moderated intervention effects on valence after correction
Affective state – arousal	LMM with 3-way Group × Time × Moderator interaction	No moderator significant after BH correction (all q >.05)	No demographic variable significantly moderated intervention effects on arousal after correction
Stigma – controllability	LMM with 3-way Group × Time × Moderator interaction	No moderator significant after BH correction (all q >.05)	No demographic variable significantly moderated intervention effects on controllability after correction
Stigma – avoidance/social distance	LMM with 3-way Group × Time × Moderator interaction	Two moderators significant after BH correction: Age: *p* =.012, q =.043 Parental status: *p* <.001, q =.002	Age and parental status significantly moderated the group × time interaction for social distance avoidance after BH correction
Stigma – punitive responses	LMM with 3-way Group × Time × Moderator interaction	No moderator significant after BH correction (all q >.05)	No demographic variable significantly moderated intervention effects on punitive responses after correction
Openness	LMM with 3-way Group × Time × Moderator interaction	No moderator significant after BH correction (all q >.05)	No demographic variable significantly moderated intervention effects on openness after correction
Therapy availability	LMM with 3-way Group × Time × Moderator interaction	No moderator significant after BH correction (all q >.05)	No demographic variable significantly moderated intervention effects on literacy about therapy availability after correction
Therapy effectiveness	LMM with 3-way Group × Time × Moderator interaction	No moderator significant after BH correction (all q >.05)	No demographic variable significantly moderated intervention effects on literacy about therapy effectiveness after correction

^1^For all comparisons, CG was defined as reference category

^2^Post hoc comparisons are not applicable if no effects are found in 1a

#### RQ1b: Differences between interventions

To assess whether the three intervention types differed from one another, Bonferroni-corrected pairwise contrasts based on estimated marginal means were conducted for the LMM outcomes, and pairwise chi-square tests with Bonferroni correction were used for self-reported website visits. For affective valence, no significant differences were found between the intervention groups at t1: IN1 vs. IN2, △ = 1.37, *p* = 1.000; IN1 vs. IN3, △ = - 3.18, *p* = 1.000; IN2 vs. IN3, △ = - 4.55, *p* = 1.000. Similarly, no significant differences between intervention groups emerged for arousal: IN1 vs. IN2, △ = - 0.73, *p* = 1.000; IN1 vs. IN3, △ = - 4.88, *p* = 1.000; IN2 vs. IN3, △ = - 4.16, *p* = 1.000.

For stigma-related outcomes, no significant pairwise differences between intervention groups were observed for controllability, avoidance/social distance, perceived dangerousness toward children and adolescents, openness, therapy availability, or therapy effectiveness after Bonferroni correction. For punitive responses, no significant pairwise differences were found at t1. One cross-timepoint contrast was significant, indicating a difference between IN2 at t1 and IN1 at t2, △ = - 0.411, *p* = .004. This finding suggests a differential rebound pattern in punitive responses at follow-up, but it should be interpreted cautiously given its isolated nature and the broader absence of consistent intervention differences. No significant pairwise differences were observed for self-reported visits to *Troubled Desire*.

#### RQ1c: Potential adverse or ethical implications

Potential adverse effects were examined by inspecting within-group changes and effect directions across affective, attitudinal, cognitive, and behavioral outcomes. For affective valence, the control condition showed an increase from t0 to t1, △ = + 13.3, 95% CI [+ 5.3, + 21.3], whereas all intervention groups showed decreases in valence: IN1, △ = - 35.5, 95% CI [- 43.7, - 27.3]; IN2, △ = - 34.5, 95% CI [- 42.7, - 26.3]; IN3, △ = - 30.8, 95% CI [- 38.9, - 22.6]. Thus, exposure to the CSA/M-related intervention materials was associated with a more negative immediate affective state.

For arousal, the control condition showed an increase in arousal-grid scores, △ = + 18.3, 95% CI [+ 11.0, + 25.7]. Because higher values indicate lower arousal, this reflects reduced emotional activation in the control group. The intervention groups showed only small and nonsignificant increases in arousal-grid scores: IN1, △ = + 5.7, 95% CI [- 1.8, + 13.2]; IN2, △ = + 3.3, 95% CI [- 4.2, + 10.9]; IN3, △ = + 1.1, 95% CI [- 6.4, + 8.6]. Relative to the neutral control condition, the intervention materials therefore appear to have induced stable activation levels.

For stigma-related outcomes, there was no consistent evidence of adverse effects in the form of increased stigma. Controllability showed no practically relevant changes. Avoidance/social distance increased slightly in the intervention groups from t0 to t1, with estimates of △ = + 0.081 for IN1, △ = + 0.086 for IN2, and △ = + 0.103 for IN3, while the control group showed △ = - 0.017. Although these changes were statistically detectable in some contrasts, their magnitude was negligible. Punitive responses decreased from t0 to t1 in all groups, including the control group, and perceived dangerousness toward children and adolescents decreased in IN1 and IN2. Dangerousness toward adults, fear, pity, and anger showed only small descriptive changes without evidence of practically meaningful adverse effects.

For cognitive and behavioral outcomes, no clear adverse effects were observed. Openness decreased across all groups, but the magnitude of change did not differ significantly by intervention condition. Therapy availability showed a small overall increase over time, while therapy effectiveness showed a small decrease over time; neither outcome showed a significant group × time interaction. Intention to visit Troubled Desire and self-reported visits did not differ significantly between groups. Taken together, the results indicate short-term negative affective reactions to the CSA/M-related materials, but no consistent evidence of increased stigma, reduced perceived treatment availability, reduced treatment effectiveness beliefs, or lower behavioral intentions.

#### RQ2a: Moderating effects

Across nine outcome domains, seven demographic moderators were tested as potential modifiers of the group × time interaction using three-way LMM interactions, with Benjamini–Hochberg (BH) correction applied within each domain (k = 7 per domain, 63 tests total). For eight of the nine outcomes, no moderator survived BH correction: this included valence (nominally: age *p* = .017, q = .105; city size *p* = .042, q = .105; education *p* = .045, q = .105), controllability (nominally: city size *p* = .022, q = .151; language *p* = .043, q = .151), and dangerousness toward children/adolescents (nominally: language *p* = .013, q = .073; city size *p* = .021, q = .073). The sole exception was “social distance/avoidance”, where both age (*p* = .012, q = .043) and parental status (*p* < .001, q = .002) significantly moderated the intervention effect over time after BH correction; gender showed a further nominal trend (*p* = .059, q = .137). Given that the main group × time interaction for social distance avoidance itself carried a small effect (η^2^p = .006), any three-way moderation effect necessarily accounts for an even smaller share of variance and should be classified as negligible to small in magnitude. All remaining outcomes (arousal, punitive responses, openness, therapy availability beliefs, and therapy effectiveness beliefs) showed no moderation effects even at the uncorrected level (all raw *p* = .098).

#### RQ2b: Impact of intervention on dropout

Of 2,019 participants at baseline, 1,115 completed t2 and 904 dropped out overall. By group, dropout was: CG: 228/518 (44.0%); IN1: 218/500 (43.6%); IN2: 240/498 (48.2%); IN3: 218/503 (43.3%). The chi-square test indicated no significant association between intervention group and dropout, X^2^(3) = 3.17, *p* = .366, with a small effect (Cramér’s V = .04). Assumptions were met (minimum expected count = 222.98; 0/8 cells < 5). Baseline comparisons between completers and dropouts revealed FDR-significant differences on age (*M*_completers_ = 44.83, *SD* = 12.60 vs. *M*_dropouts_ = 42.63, *SD* = 12.64; *t*(1929) = 3.90, *p* FDR < .001, *d* = 0.18) and language (*V* = 0.18, *p* FDR < .001), with Spanish-speaking participants showing higher dropout rates. Completers and dropouts did not differ on any baseline psychological outcome variable after FDR correction (all *p* FDR = .09). The largest categorical difference was observed by language of participation, with Spanish-speaking participants showing substantially higher dropout (53.4%) than Portuguese-speaking participants (Portugal; 35.8%), *OR* = 2.06, *V* = 0.175, *p* FDR < .001.

## Discussion

The present study investigated the effects of three communication interventions – educational messaging, humanizing with prevention focus, and humanizing with empathy focus – on affective, cognitive, attitudinal, and behavioral outcomes related to CSA/M prevention. The primary finding was a significant short-term shift in affective responses: participants who received any of the three intervention messages reported more negative affective valence and slightly elevated arousal immediately after exposure, relative to a control group that received a neutral diabetes message. Critically, no significant group × time effects emerged for any of the stigma dimensions (controllability, dangerousness, social avoidance, punitive attitudes, fear, anger, or pity), literacy about available therapy, openness, or self-reported behavioral intentions. In the sections below we interpret these findings, situate them within existing theoretical and empirical literature, and discuss implications and limitations.

### Affective effects

Exposure to CSA/M-related intervention content produced a decrease in positive affective valence (medium effect) and a slight increase in arousal (small effect) immediately after viewing. These shifts were consistent across IN1, IN2, and IN3. The control condition showed a small increase in positive affect and a slight decrease in arousal after reading about diabetes management. This pattern indicates that any engagement with CSA/M-themed content – regardless of message framing – evokes an immediate affective response that differs from exposure to neutral health content. Critically, post-intervention affective state showed no predictive value for dropout, suggesting that affective activation did not drive disengagement. Whether such activation promotes subsequent attitude elaboration or, conversely, interferes with message processing through avoidance or reactance, cannot be determined from these data alone. A further interpretive constraint is that all three intervention groups were exposed to the same video depicting a man watching CSAM before being interrupted, whereas the control group viewed an unrelated video. The observed affective differences therefore likely reflect the salience of the CSAM topic or the implied criminal behavior in the video rather than the specific educational or humanizing message components.

### Null findings on stigma, cognition, and behavioral outcomes

None of the three interventions produced significant changes in any of the stigma dimensions, cognitive indicators, or behavioral outcomes assessed. These null findings are the central empirical contribution of the study and warrant careful, multi-level interpretation.

First, the distributions observed across stigma-related measures showed pronounced ceiling effects and, in some cases, bimodal response patterns, particularly for the dimensions of dangerousness and controllability. These response characteristics indicate that many participants already held highly stigmatizing attitudes prior to the intervention. When participants already endorse maximal attitudes, the statistical room for detecting a reduction is minimal: a genuine intervention effect may be invisible to the measurement instrument. The bimodal distributions, in turn, suggest attitudinal polarization: while one subgroup expressed strong rejection and moral condemnation, another displayed more differentiated or empathetic views. Overall, these findings demonstrate that stigma toward individuals at risk is not normally distributed but clustered around the extremes of the scale. This poses both methodological and conceptual challenges for evaluating intervention effects, as high baseline scores near the upper scale limit imply that the true level of stigma may lie beyond the measurable range. Consequently, changes induced by brief interventions are unlikely, given the deeply entrenched and resistant nature of such attitudes.

Second, the intervention deliberately combined short video and text elements to mirror typical media consumption patterns. Attitudes toward individuals with a sexual interest in children represent some of the most strongly held and morally sanctioned negative judgments in public discourse [44], and evidence from the anti-stigma literature indicates that such attitudes are unlikely to shift from single brief exposures. However, Fix et al. [13] note that, with reference to Gough et al. [17], the use of social media outlets like Twitter to promote public health campaign messaging has demonstrated preliminary feasibility and efficacy in changing attitudes and behaviors. Nevertheless, studies employing brief interventions, e.g., 5- to 12-min videos or short written materials, have reported only small to medium effect sizes (d = 0.11–0.79), with limited practical relevance (often representing less than a one-point change on Likert-type scales) [22, 32, 38]. In contrast, larger effects tend to emerge from more intensive interventions, such as extended contact sessions lasting around two hours (d = 0.76–1.49; [18]) or semester-long educational courses, which yield medium to large effect sizes [24, 56]. These findings suggest a possible dose–response relationship: brief interventions may evoke momentary emotional activation but are generally insufficient to produce lasting cognitive or behavioral change. Comparable short-format messaging has been used in deterrence and warning message studies [42, 43], where the interventions demonstrated measurable effects on behavior. However, those messages were usually embedded within highly contextualized environments, such as adult content platforms, which likely heightened their salience and immediacy.

Third, the equivalence across the three intervention formats raises questions about mechanism specificity. Because all three conditions shared the same video stimulus, any text-level persuasive differences between educational and humanizing frames may have been overwhelmed. The distinct theoretical mechanisms hypothesized to drive IN1 and IN2 produced statistically indistinguishable outcomes. This could mean either that all three mechanisms operated equally (all ceiling-constrained), that the shared video neutralized text-level differences, or that none of the intended mechanisms was engaged.

### Adverse effects and ethical considerations

Although the interventions elicited short-term emotional discomfort, the interventions did not produce significant increases in any stigma dimension, no attitudinal rebound, and no condition-specific dropout These findings provide preliminary evidence that brief CSA/M-related communication messages did not produce measurable short-term increases in stigma-related outcomes in this general population sample which is particularly relevant given that previous studies have reported potential backfire effects of psychoeducational materials. For example, Harper et al. [22] observed that an informative condition initially increased deviance perceptions toward individuals with a sexual interest in children, while Jara and Jeglic [29] found that psychoeducational interventions sometimes led to heightened negative attitudes rather than reducing them. However, the absence of increases in measured stigma indicators does not constitute a comprehensive demonstration of ethical acceptability.

### Drop-out and attrition

Of the 2,019 participants at baseline, 1,115 completed the posttest (t2), resulting in an overall attrition rate of 44.8%. Dropout rates were comparable across conditions, and a chi-square test confirmed no significant association between intervention group and drop-out. Thus, participation and retention were not differentially affected by message type. This finding is notable given the sensitive and potentially discomforting nature of CSA/M-related content. Although participants who completed the study tended to be older and more likely to be Portuguese-speaking than those who dropped out, completers and dropouts did not differ on any baseline measure of psychological or behavioral variable after correction for multiple comparisons, reducing concern that differential attrition introduced systematic bias into the longitudinal outcome analyses. The higher dropout rate observed among Spanish-speaking participants may reflect differences in cultural norms surrounding engagement with sensitive topics, though this explanation remains speculative. Notably, language of participation did not moderate intervention effects. 

### Cultural context and generalizability

The study was conducted in Spain and Portugal – two countries with comparatively limited long-term public discourse or media infrastructure around CSA/M prevention. This context was selected to minimize prior-exposure effects and to extend the empirical evidence base to non-English-speaking general populations, where most prior trials have not been conducted.

A recurring limitation noted in previous research concerns the composition of study samples, which have often consisted primarily of young, female, higher-education participants [18, 21, 24, 32, 33, 35, 56]. This restricted demographic focus limits the generalizability of findings and hampers insights into how different population groups respond to anti-stigma communication.

Christiansen et al. [7] explicitly note that “when developing educational programs that target stigmatization, it is essential to consider the cultural aspect. Both the national and cultural context shapes the extent of stigma and stereotypes through, e.g., social organization, the legal and cultural system.” Similarly, Langvik et al. [30], reported notable differences between U.S. and Norwegian samples, particularly regarding public endorsement of treatment versus imprisonment for CSAM offenders. These findings underscore that while the language-related effects in the present study were minimal, cultural and contextual factors may still play an important role in shaping public perceptions and responses to prevention messaging.

### Implications

Although the intervention did not yield significant improvements across stigma, cognitive or behavioral measures, it also produced no clear evidence of short-term adverse effects on the assessed outcomes – an ethically important finding and a valuable foundation for future health communication activities in the sensitive domain of CSA/M prevention. Given the intervention’s ability to generate short-term affective responses, this format may serve as a useful starting point for capturing attention and initiating engagement. However, as the present findings indicate, such brief, single-exposure interventions are insufficient to trigger durable changes in cognition, attitudes, or behavior. Findings from the literature hint to a dose–response mechanism. Building on this, future communication strategies might consider video-based content as a gateway mechanism; a means of emotional activation that can lay the groundwork for deeper, repeated, or more cognitively demanding engagement. As several researchers have emphasized, sustained impact is unlikely without prolonged or repeated exposure. For instance, Lawrence and Willis [32] call for repeated interventions to promote sustained attitudinal changes, while Harper et al. [22] recommend longer interventions to see whether deeper or more developed arguments enhance the effects. Similarly, Jahnke, Philipp, and Hoyer [28] suggest integrating online interventions as components within broader educational frameworks because attitudes that have developed over a long time tend to be stable. Future campaigns may benefit from greater intensity and repeated exposure since these are essential for attitude change.

Moreover, intervention design should take into account current media consumption patterns. Given the prevalence of short-form content, especially on social media platforms, future stigma reduction strategies may benefit from repetitive, episodic exposure rather than isolated, one-off campaigns. A strategically designed series of concise, emotionally resonant, and educationally grounded messages, delivered repeatedly across platforms, could increase the likelihood of sustained cognitive engagement and, over time, meaningful attitude change.

The findings of this study offer a concrete and actionable implication for public health campaigns, even in the absence of detectable attitude change: brief, carefully designed messages may draw public attention to CSA/M without evidence of increased stigma. This is a critical insight, as preventing unintended stigmatizing effects represents a central ethical and practical threshold for any population-level intervention in this domain. The findings suggest that short-form communication formats they may function as a feasible entry point into broader prevention strategies – raising awareness, initiating engagement, and preparing audiences for more intensive or elaborated messaging.

Thus, the most immediate translational pathway emerging from this study is the development of short, ethically robust communication modules that can be scaled across digital platforms. These modules can be used to sensitize the public to the topic, signal the legitimacy of preventive engagement, and ultimately support a layered communication strategy in which brief attention-grabbing messages anchor more comprehensive educational or therapeutic resources.

### Limitations

Several limitations of the present study should be acknowledged. First, the comparability of the three intervention conditions was limited due to non-standardized formats. Although all conditions included both a video and a textual component, variations in length and information density may have influenced participants’ emotional or cognitive engagement. Since all intervention conditions shared the same CSA/M-related audiovisual stimulus while the control condition differed substantially in thematic and emotional salience, the observed affective effects cannot be cleanly attributed to the educational or humanizing framing components themselves.

Second, group assignments were conducted using simple randomization rather than stratified randomization. Given the large sample size (> 1,000), this approach was chosen to optimize efficiency, though it may have introduced imbalances in subgroup distribution that could have affected moderation analyses.

Third, a potential priming effect must be considered, as participants completed baseline measures on stigma and related constructs immediately prior to the affective state rating and exposure to the intervention materials. Fourth, the study relied exclusively on self-report measures and did not include objective behavioral data. For example, actual engagement behavior such as website visits were not tracked. This limits the ability to draw conclusions about real-world behavioral impact beyond self-reported intentions.

A further limitation concerns the generalizability of findings to population groups with limited digital literacy or restricted access to online platforms. As the study relied on an online survey design and brief multimedia interventions, individuals who are less familiar or less comfortable with digital technology may be underrepresented. This is relevant given that prevention campaigns ideally reach across demographic groups, including older adults or individuals in digitally marginalized contexts. Notably, attrition analyses indicated that older participants were less likely to drop out than younger participants. This suggests that older participants showed greater study commitment once enrolled, which may reflect the motivational profile of individuals who engage with online research.

One could argue that the online format closely mirrors the real-world environment in which modern prevention campaigns increasingly disseminate information, particularly through social media and short-form digital content. Thus, while online methodology imposes certain constraints, it also reflects the natural ecology of contemporary health communication. Nonetheless, future work should consider complementary offline strategies to ensure that prevention messaging is accessible to audiences with varying levels of digital engagement.

An additional conceptual limitation concerns the terminology used in the intervention materials. Although the manuscript consistently uses the term child sexual abuse material (CSAM), the intervention referred to “so-called child pornography” because this expression remains more widely recognized in general public discourse and was therefore considered more accessible in a brief public communication context. However, terminology itself constitutes an important component of public education and stigma-related framing. The study did not assess whether participants understood the distinction between “child pornography” and CSAM terminology, nor whether terminology influenced message reception. Future research should examine more directly how terminology affects public understanding, perceived severity, stigma, and prevention-related communication outcomes.

Cultural context may limit the transferability of findings. The study was conducted in Spain and Portugal – countries with comparatively little long-term public discourse or media visibility around CSA/M prevention initiatives. Spain and Portugal were selected to reduce the likelihood of pre-exposure bias, however, it restricts straightforward generalization to contexts with different legal frameworks, media histories, or cultural narratives surrounding CSA/M. The differential retention observed between both countries – with Spanish participants dropping out at higher rates despite representative quotas – further illustrates that cultural context may shape not only how participants respond to intervention content but also their willingness to engage. This underscores the importance of culturally adapted engagement strategies in future cross-national prevention research.

Importantly, the present study did not directly measure empathy, dehumanization, perceived humanity, or narrative transportation. Consequently, the proposed psychological mechanisms underlying the humanizing interventions remain theoretically inferred rather than empirically demonstrated. Future studies should directly assess these mediating constructs to determine whether affective activation translates into meaningful changes in stigma-related cognition or behavioral intentions.

## Conclusions

This study provides preliminary evidence that brief CSA/M-related communication interventions can elicit short-term affective responses without measurable increases in stigma-related outcomes. Although no significant changes in stigma, attitudes, or behavioral intentions were observed, the findings confirm that short-form interventions can serve as an initial point of contact for capturing attention and initiating engagement with the topic. To achieve meaningful and lasting attitude change, future efforts should build on this format through repeated exposure, narrative depth, and integration into broader educational or clinical contexts. Crucially, translating empirical knowledge into publicly resonant messages remains a pressing challenge. As Fix et al. [13] argue, “new ways of translating this evidence base are needed to improve the public’s understanding of CSA – why it happens, why it matters, and how it can be prevented.” (p. 3) Strategic testing of explanatory metaphors and empirically validated framing approaches represents a promising next step in this process. Moreover, the dissemination of CSA prevention messaging must extend and include diverse channels, embedded framing guidance, and strategic partnerships with organizations in the child protection field. As Fix and colleagues emphasize, such coordinated efforts can empower the field to “speak with a more strategic and consistent voice,” thereby enhancing public understanding and support for effective, evidence-based prevention policy.

## Data Availability

The datasets generated and analyzed during the current study are not publicly available due to the sensitive nature of the topic and ethical restrictions related to participant anonymity and data protection. Anonymized data are available from the corresponding author on reasonable request for research purposes.

## Abbreviations

ACE: Adverse Childhood Experiences
ANOVA: Analysis of Variance
ATS: ANOVA-Type Statistic
CG: Control Group
CI: Confidence Interval
CLMM: Cumulative Link Mixed Model
CSA: Child Sexual Abuse
CSAM: Child Sexual Abuse Material
CSEM: Child Sexual Exploitation Material
DSM-5: *Diagnostic and Statistical Manual of Mental Disorders*, Fifth Edition
FDR: False Discovery Rate
IN1: Intervention 1 – Educational Message
IN2: Intervention 2 – Humanizing Message (Empathy Focus)
IN3: Intervention 3 – Humanizing Message (Prevention Focus)
LMM: Linear Mixed-Effects Model
OR: Odds Ratio
RQ: Research Question
SCM: Sterotype Content Model
SD: Standard Deviation
SE: Standard Error
WHO: World Health Organization

## Footnotes

1 Data quality was ensured by: 1. a manipulation check after the intervention exposure, consisting of two multiple-choice items that referred to group-specific content (“The health information campaign I saw included information about…”, “The health information campaign I saw included a placeholder link to a website.”), 2. the analysis of response times to detect inattentive or invalid responses. Specifically, time spent on the first eight pages of the survey was examined. Participants who remained on at least three of those pages for less than 5 s were considered inattentive and were excluded.

2 Two extreme outliers were excluded from the descriptive statistics reported here. However, these cases were retained in the full analytic dataset because there were no indications of invalid responding or data quality concerns.
